# SARS-CoV-2–encoded ORF8 protein possesses complement inhibitory properties

**DOI:** 10.1016/j.jbc.2023.102930

**Published:** 2023-01-20

**Authors:** Jitendra Kumar, Saurabh Dhyani, Prateek Kumar, Nishi Raj Sharma, Surajit Ganguly

**Affiliations:** 1 Department of Molecular Medicine (DMM), Neurobiology and Drug Discovery (NDD) Laboratory, Jamia Hamdard, New Delhi, India; 2 School of Biosciences and Bioengineering, Indian Institute of Technology Mandi, VPO Kamand, Himachal Pradesh, India

**Keywords:** covid-19, SARS-CoV-2, complement, innate immunity, alternative pathway, AP, alternative pathway, Co-IP, coimmunoprecipitation, FB, Factor B, FD, Factor D, FH, Factor H, FI, Factor I, H-bond, hydrogen bond

## Abstract

Hyperactivation of the complement system, a major component of innate immunity, has been recognized as one of the core clinical features in severe covid-19 patients. However, how the virus escapes the targeted elimination by the network of activated complement pathways still remains an enigma. Here, we identified SARS-CoV-2–encoded ORF8 protein as one of the major binding partners of human complement C3/C3b components and their metabolites. Our results demonstrated that preincubation of ORF8 with C3/C3b in the fluid phase has two immediate functional consequences in the alternative pathway; this preincubation inhibits factor I–mediated proteolysis and blocks factor B zymogen activation into active Bb. ORF8 binding results in the occlusion of both factor H and factor B from C3b, rendering the complexes resistant to factor I–mediated proteolysis and inhibition of pro-C3-convertase (C3bB) formation, respectively. We also confirmed the complement inhibitory activity of ORF8 in our hemolysis-based assay, where ORF8 prevented human serum–induced lysis of rabbit erythrocytes with an IC_50_ value of about 2.3 μM. This inhibitory characteristic of ORF8 was also supported by in-silico protein-protein docking analysis, as it appeared to establish primary interactions with the β-chain of C3b, orienting itself near the C3b CUB (C1r/C1s, Uegf, Bmp1) domain like a peptidomimetic compound, sterically hindering the binding of essential cofactors required for complement amplification. Thus, ORF8 has characteristics to act as an inhibitor of critical regulatory steps in the alternative pathway, converging to hasten the decay of C3-convertase and thereby, attenuating the complement amplification loop.

Detection and elimination of viral pathogens by the host complement system has been known since 1930 (1). Though the tightly controlled complement pathways are regarded as major innate defense mechanism of the host, accidental dysregulation of any of the components leading to hyperactivation of the complement system can cause devastating damage to the host tissues (2). This tissue damaging complement activation has been demonstrated to be the hallmark of pathophysiology in the large section of covid-19 patients (3, 4). Emerging data implicate both the spike and nucleocapsid proteins of SARS-CoV-2 in the activation of the lectin as well as the alternative complement pathways (5, 6). Thus, complement activation in covid-19 patients appears to be more of a bystander effect rather than by design to gain advantage by the virus in the host. So, a major question remained unanswered so far about how SARS-CoV-2 escapes the complement system surveillances despite its robust activation.

In order to counter the complement-induced host defense system, viruses have also evolved unique strategies to escape this surveillance network (7, 8, 9). Frequent instances are documented where the viruses have adopted molecular mimicry by encoding orthologs of the complement family of proteins or co-opting the host complement system to gain evolutionary advantage (10, 11). This led us to investigate whether similar strategies are also adopted by SARS-CoV-2 to bypass activated complement pathways. During activation, complement alternative pathway (AP) converge on cleaving the complement component C3 into C3a and C3b fragments by a functional C3-convertase (C3bBb) complex. Activation of factor B (FB) by Factor D (FD) is a prerequisite for C3-convertase formation. C3b acts as a major opsonin tagging the pathogen surface and promoting assembly of the convertases, leading to its amplification and targeted clearance of the pathogen (12). Factor I (FI)-mediated cleavage of C3b acts as a critical negative-feedback regulatory arm of the AP. Here, we report the interaction of SARS-CoV-2–encoded ORF8 protein with C3b and unravel the molecular mechanism of this binding. The functional significance of this protein–protein interaction has also been demonstrated.

## Results

**Figure 1:**
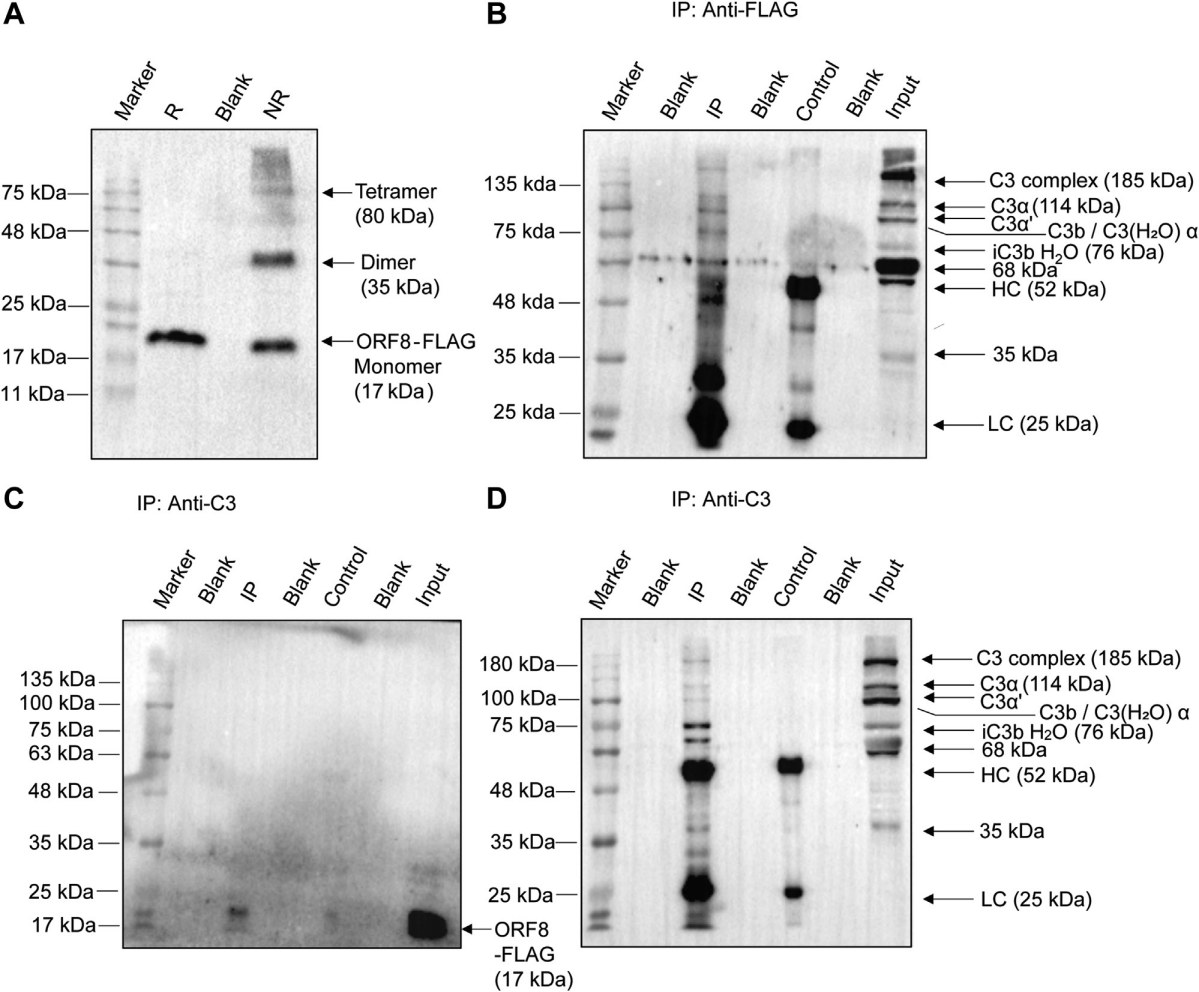
**Expression of SARS-CoV-2 ORF8 and interaction with complement C3 and its metabolites by Coimmunoprecipitation.***A*, multimeric forms of ORF8-FLAG–tagged protein, expressed in transfected HepG2 cells and immunoblotted using anti-FLAG antibody in lysates prepared under reduced (R) *versus* nonreduced (NR) condition. Monomers (17 kDa) in standard Laemmli buffer, oligomerizes in NR condition. Co-IP from FLAG-ORF8–transfected HepG2 cells as indicated in panels. *B*, IP with mouse-anti-FLAG antibody and detection on blot by rabbit anti-C3 antibody against C3dg/TED domain encompassing fragment (amino acid 1000–1326 of human C3); *C*, IP with rabbit anti-C3 antibody and detection on blot with anti-FLAG. *D*, reprobing of the stripped-blot in C panel with rabbit anti-C3. Anti-C3 recognizes epitopes in C3, C3b, iC3b, and C3c fragments, as indicated. Antibody light chain (LC) and heavy chain (HC) are also shown. For control lanes (anti-rabbit IgG antibody), IP lanes (target antibodies), and input lanes (lysate), 15% equivalent and 5% of total input samples were loaded, respectively. Data shown is representative image of three independent experiments. The colorimetric images for prestained markers (Blueye prestained protein marker; GeneDireX) in panels and the Chemiluminescence images from the corresponding immunoblots were captured and merged using Image Lab software (Bio-Rad), associated with ChemiDoc XRS+ imaging system (Bio-Rad) to generate the representative images.

To investigate whether ORF8 interacts with complement C3 or any of its cleaved metabolites of the AP, we used a flag-tagged codon-optimized construct of SARS-CoV-2 ORF8 as a bait. The ORF8-Flag construct was expressed in HepG2 cell line as a 17 kDa protein as expected from the calculated size (Fig. 1*A*). In nonreducing condition, a population of expressed protein migrated as dimer at 34 kDa and other multimeric forms (Fig. 1*A*), confirming the ability of the ORF8 to form dimer and tetramer as reported earlier (13). Thus, after validating the expression of ORF8 in HepG2 cells, we interrogated its interaction with complement C3 and its metabolites using coimmunoprecipitation (Co-IP; Fig. 1, *B*–*D*) assays. Anti-C3 antibody immunoprecipitated ORF8-Flag protein (detection by anti-Flag antibody on blot) from HepG2 cell lysate made from the cells transfected with the ORF8-Flag (IP lane, Fig. 1*C*). Reprobing the same blot with anti-C3 antibody (Fig. 1*D*) surprisingly revealed detection of the multiple C3-metabolites. The anti-C3 antibody (raised against recombinant fragment of 1000–1326 residues on α chain of human C3) used for this work has common epitope(s) on native C3, C3b, iC3b, and C3c. This enabled it to detect cleaved fragments generated from C3 having sizes ranging from 114 kDa (α chain) to 35 kDa (C3d-like fragment) (input lanes, Fig. 1, *B* and *D*). On reversing the IP antibody to anti-Flag, not only the native C3 protein was pulled down by Co-IP but its cleaved metabolites C3b, iC3b, and C3c also, as confirmed in Figure 1*B*. Thus, it appeared that ORF8 has the ability to interact with the native complement component C3, along with its cleaved metabolites C3b (α′) and other smaller complexes derived from cleavage of α-chain [(14, 15); Fig. 1].

**Figure 2:**
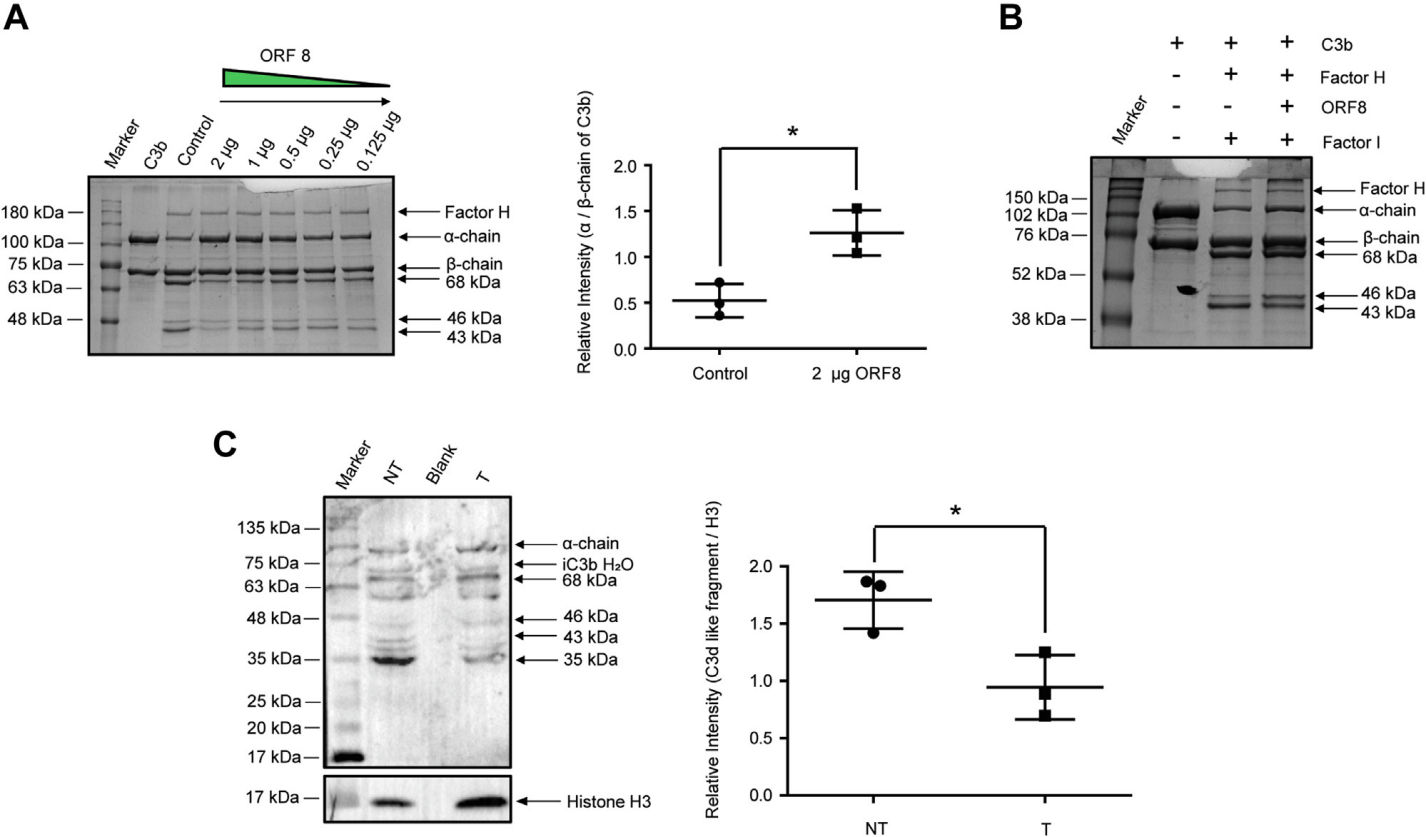
**ORF8 inhibits FI-mediated cleavage of C3b α-chain.***A*, C3b and His-ORF8 were preincubated as indicated, prior to the addition of FH and FI. The relative intensity of α-chain of C3b (cleaved into 68, 46, and 43 kDa fragments by FI) with respect to uncleaved β-chain, calculated in the absence (control) and presence of His-tagged ORF8 (2 μg) by densitometry on Coomassie blue–stained gel is plotted as scatter plot using for comparison. *B*, C3b-FH preincubation prior to the addition of ORF8. No inhibitory effect of ORF8 on FI activity. *C*, ORF8-FLAG transfected (T) in HepG2 cells attenuates C3b degradation by endogenous FI in HepG2 cells as compared to mock-transfected (NT) cells. Comparison of the relative intensity of 35 kDa (C3d-like fragment) band, detected by rabbit anti-C3 analyzed by densitometry, is an indicator of endogenous serine-protease (including FI) activity. Blueye prestained protein marker (GeneDireX) was used for panels (*A*) and (*C*), whereas Rainbow maker (Amersham) was used for panel (*B*). The differences in intensities of the protein bands, as indicated in (*A*) and (*C*) with respect to histone H3 as loading control, were calculated from Scatter plots generated by GraphPad Prism software from mean ± SD values of three independent experiments (Two-tailed unpaired *t* test; ∗*p* < 0.05). FH, Factor H; FI, Factor I.

Next, as a proof of concept, we investigated the functional significance of the interaction between ORF8 and C3b. For this, we used a competition assay with FI for its ability to cleave C3b to produce iC3b and C3c, in the presence or absence of ORF8. FI is known to hydrolyze the three conserved Arg-Ser (R-S) cleavage sites of C3b CUB (C1r/C1s, Uegf, Bmp1) domain in the presence of another cofactor, factor H (FH) (16). In a reconstitution experiment *in vitro*, purified C3b was preincubated separately with increasing molar concentration of recombinant ORF8, followed by the addition of FH. The cleavage of C3b was initiated with the addition of FI, and the degradation of C3b (α chain 114 kDa) was monitored for 7 min at 37 °C. The activity of FI was determined by monitoring the generation of two major α-chain–degraded fragments of iC3b (68 kDa) and C3c (46/43 kDa) from C3b on denatured polyacrylamide gels. The presence of ORF8 inhibited the generation of iC3b and C3c fragments as compared to the control (Fig. 2*A*), where no ORF8 was added. Maximum inhibition of proteolysis activity of FI was achieved in the presence of 2 μg of ORF8. Quantification of band intensities revealed significant accumulation of α-chain of C3b (about 2.5-fold using unchanged β-chain intensity as reference; Fig. 2*A*), with a concomitant decrease of the iC3b fragment (68 kDa), though 0.125 μg of ORF8 was adequate to inhibit FI at varying extent. However, when C3b was incubated with FH before ORF8 was added, no effect of ORF8 (2 μg) on FI-mediated C3b proteolysis was observed (Fig. 2*B*). This appeared to suggest that FI-mediated proteolysis required ORF8 to bind sequentially before FH binding and it had no direct inhibitory effect on FI activity, perhaps signifying its lower affinity for C3b binding than FH.

Thus, to confirm the impact of ORF8 expression on C3b processing in cellular environment, we transfected ORF8 (flag-tagged construct) in HepG2 cell line and monitored the effect of its expression on the generation of cleaved-C3b products as a function of competition with the endogenous cellular FI activity. Accordingly, the cell lysates were prepared and immunoblotted to compare the intensity of the FI-generated smaller cleaved products like C3c and C3d fragments, in transfected *versus* mock-transfected cells (Fig. 2*C*). Comparative immunoblot analyses revealed that in the ORF8-transfected cells, there was a significant decrease in the generation of C3b-cleaved products of sizes ranging from 46 kDa to 35 kDa (Fig. 2*C*) with respect to the mock-transfected controls. The intensity of the final cleaved fragment, the C3d-like 35 kDa protein band was reduced by about 2-fold (Fig. 2*C*) as compared to the control. Though generation of such smaller C3 fragments by endogenous serine-proteases (like trypsin) from the three conserved R-S sites on the CUB domain other than FI cannot be ruled out [(17), Fig. S1], this result appeared to be consistent with our reconstitution experiment (Fig. 2*A*), suggesting that the association of ORF8 made C3b more resistant to proteolysis.

**Figure 3:**
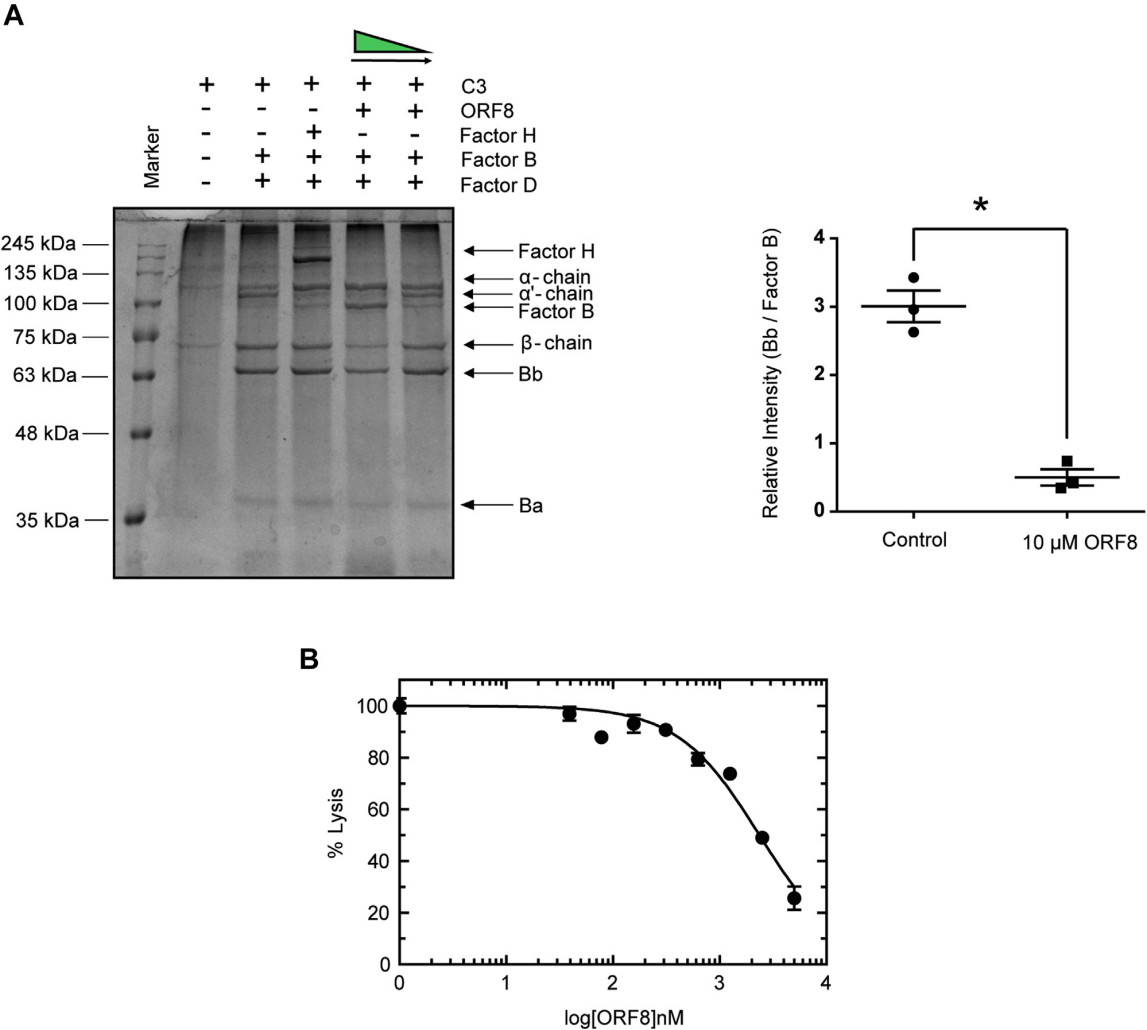
**ORF8 inhibits human AP complement activity.***A,* In C3-convertase fluid phase assay, α-chain of C3 is cleaved to α′ by activated Bb (lane 3) as described in the text. The proteolysis of α to α′ and conversion of FB (inactive form, 93 kDa) to Bb (active form, 63Kda), by FD-mediated removal of Ba (30 kDa), are maximally inhibited in the presence of 10 μM of ORF8 (lane 5). The reactants of the representative assay are shown above each lane of a Coomassie blue–stained 10% SDS-PA gel image, with lane 1 being the marker lane (lowest marker size is 25 kDa, Blueye prestained protein marker). FH in lane 4 inhibits α′ formation but not FB activation. The differential FB activation, represented by Bb/FB (lane 3 *versus* lane 5 plot), is calculated from data obtained by densitometry from three independent experiments followed by two tailed unpaired *t* test (∗*p* < 0.05). *B*, ORF8 inhibits human serum–induced hemolysis of rabbit erythrocytes. The normal human serum–induced (in absence of ORF8) lysis of rabbit RBC (1 × 10^9^/ml) was normalized to 100%. The inhibition of hemolysis by ORF8 at concentration range (39 nM to 5000 nM) was calculated with respect to lysis in the absence of ORF8. The data obtained was fit into a dose-response inhibition curve with variable slope using GraphPad Prism software. IC_50_ of ORF8-mediated inhibition of hemolysis was determined to be about 2.3 μM from the plot. Details have been provided in the supporting information. AP, alternative pathway; FB, Factor B; FD, Factor D; FH, Factor H.

Since ORF8 rendered C3b resistant to FH-FI–mediated proteolysis, we examined further to test whether ORF8 can compete with FB recruitment, another major serine protease zymogen whose activation is critical to trigger AP cascade. Binding of FB with surface-attached C3b or fluid-phase C3 (H_2_O) is a prerequisite step in its activation by FD (18, 19, 20, 21). We performed fluid-phase C3-convertase assay using purified FB, FD, and C3 to determine whether ORF8 can restrain FB locked in the proenzyme state (Fig. 3*A*). Thus, protection of FB from cleavage into Bb and Ba would be considered an inhibition of FD-mediated activation and hence, detrimental to assembly of a functional C3-convertase. On analyzing the protein profiles of the convertase assay products resolved by SDS-PAGE, we found a distinct increase in intensity of the FB protein band (93 kDa) with a concomitant decrease in the cleaved activated form, Bb (63 kDa) in the presence of ORF8 in molar excess as compared to the sample without or at low concentration of ORF8 (Fig. 3*A*). The ratio of the activated fragment Bb to zymogen FB is reduced drastically (about 6-fold) in the presence of 10 μM ORF8 (lane 5, Plot in Fig. 3*A*), suggesting that ORF8 has the ability to rescue FB from FD-mediated activation. Moreover, the absence of α' (alpha-prime) protein band in the lane 5, where ORF8 was added in molar excess (Fig. 3*A*), confirmed the inhibition of the cleavage of α-chain of native C3 to α' of C3b by removal of C3a peptide. Unlike ORF8, the presence of FH (lane 4, Fig. 3*A*) could not prevent Bb formation by FD but the generation of the cleavage product α′ is blocked, indicating that the inhibition is at the level of convertase activity only. Thus, taken together, ORF8 appeared to shield FB from binding to C3b/C3 (H_2_O) for activation, a prerequisite first step in pro-C3-convertase (C3bB) formation, inhibiting cleavage and activation of FB by FD, ultimately attenuating C3 α-chain cleavage. To confirm the physiological relevance of ORF8-mediated inhibition of complement activity, normal human serum–induced rabbit erythrocyte hemolysis-based assay was performed (Fig. 3*B*). Fifty percent inhibition of hemolysis was achieved at about 2.3 μM (calculated IC_50_) of purified ORF8. Thus, these results establish ORF8 to possess putative inhibitory properties of the host complement pathway.

**Figure 4:**
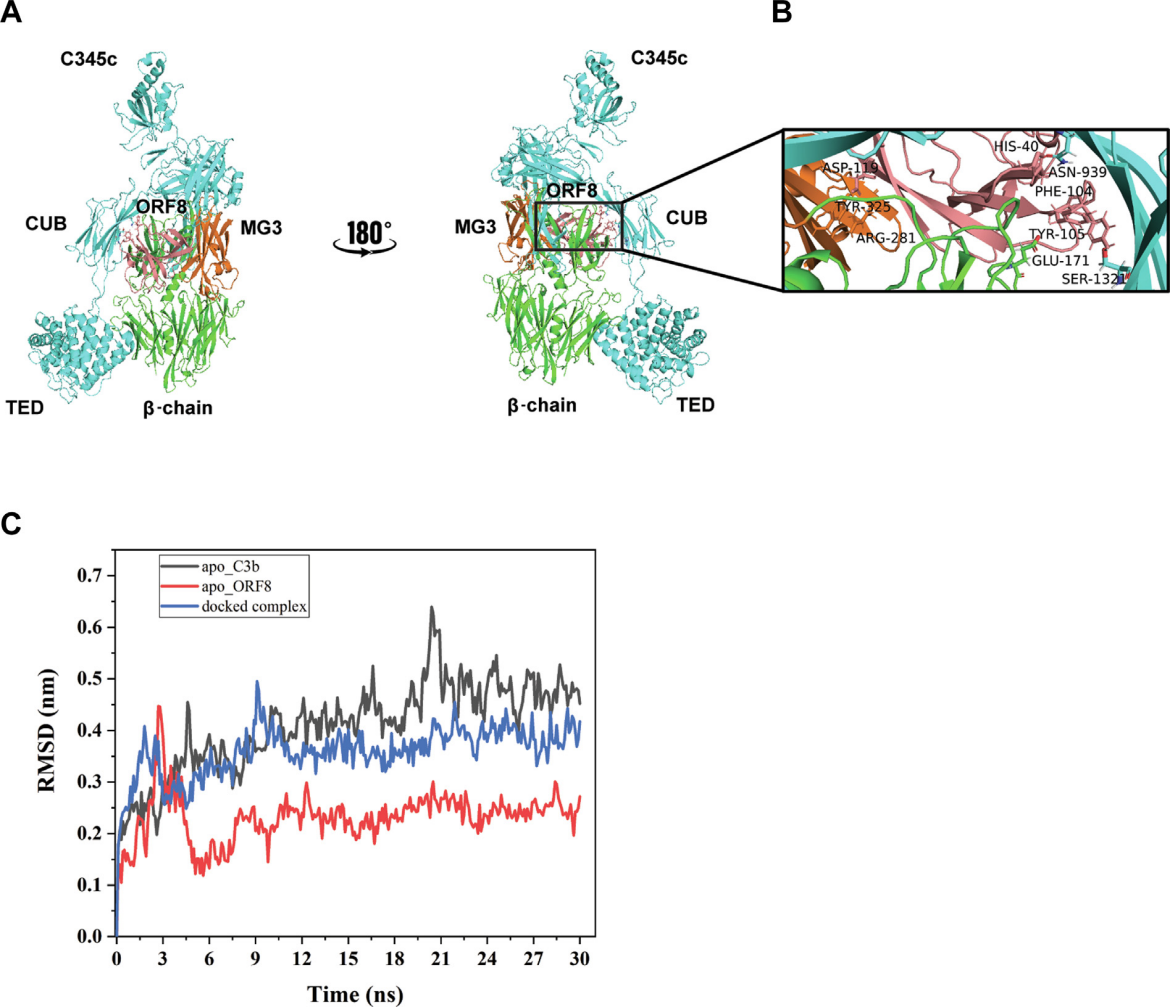
**In-silico model of C3b-ORF8 cocomplex.***A*, mini FH and FI were replaced from the structure C3b-mini FH-FI structure (PDB entry 5O32) and docked with ORF8 (PDB entry 7JTL). ORF8 (*salmon*) appears to partially occupy complement control protein (CCP) binding domain 2-3 on C3b. ORF8 is shown to make multiple contacts with the MG3 *(orange)* and MG2 domains of β-chain. *B*, zoomed view of interactions between the MG3 domain residues *(orange)*, ARG-281 and TYR-325, with ASP-119 (ORF8). Also, interactions between His-40 (ORF8)/ASP-939 (α-chain C3b) and PHE-104 (ORF8)/GLU-171 (β-chain C3b), respectively, show proximity to the second cleavage site RS (1320-1321) of CUB domain in α-chain *(cyan)* of C3b. More information in Table S1. *C*, molecular dynamics simulation analysis confirms the stabilization of the docked complex (C3b-ORF8) as compared to the apo-C3b (uncomplexed). Root Mean Square Deviation (RMSD) values (nm) generated for 30 ns at 150 mM KCl solvent system are plotted for comparison using Gromacs simulation package (details provided in supporting information). FI, Factor I; FH, Factor H.

To further support our experimental data, we used protein-protein docking analysis of C3b-ORF8 complex retrieving the published crystal structures of C3b complex ((14); PDB entry 5O32) and ORF8 (PDB entry 7JTL). First, we examined whether C3b in FH-bound form allows ORF8 to fit into the C3b-FI interface. We used the structure of C3b complex in FH-FI bound form (14) as template and replaced the FI with ORF8 monomer structure. All top five ranked structures with lowest free-energy obtained from protein-protein docking (Fig. S2) revealed no occupancy of the C3b-FI binding interface by ORF8 in the presence of FH. However, on removing FH from the complex (PDB entry 5O32), ORF8 monomer (18–121 residue; Salmon color in Fig. 4*A*) appears to occupy a binding groove partially overlapping complement control protein–binding domain 2-3 (22), on C3b (Fig. 4*A*) in one of the top ranked docked structures. Thus, our results in Figure 2, *A* and *B* appears to support the conformation of the complex structure selected (Fig. 4), suggesting that the binding of ORF8 and FH on C3b is mutually exclusive and FI activity is inhibited by ORF8 due to the occlusion of FH binding on C3b.

Further analysis of the in-silico–docked ORF8-C3b complex (Fig. 4*B*) revealed ORF8 to have major contacts with C3b using five potential hydrogen-bonds (H-bonds) (Table S1). Interestingly, three of these five C3b-interacting H-bonds are shared by two ORF8 residues, Phe-104 and Asp-119, located in the antiparallel β-strands, which has sequence resemblance with the C-terminus region of FI (Fig. S3). Moreover, the major interactions of ORF8 occur with the β-chain of C3b within the MG3 (Macroglobulin-like) domain (Arg-281, Tyr-325). These interactions perhaps allow the ORF8 to occupy the core hollow cavity in proximity to Arg-1320 and Ser-1321 on the α-chain in the CUB domain (Fig. 4*B*, inset) rendering C3b resistant to proteolysis. In addition, molecular dynamics simulation analysis (Fig. 4*C*) indicated that the complex formation appeared to stabilize C3b as compared to the free C3b (apo-form), favoring the interaction with ORF8 in simulated physiological conditions. Lower RMSD values (in nm) for the complexed-C3b confirmed the stabilization of C3b when docked with ORF8 for at least 30 nanoseconds.

Overall, the experimental results appear to be in agreement with our molecular docking results which tend to suggest that the possible binding of ORF8 with C3b occurs both with the β-chain and α-chain, strategically interfering with the recruitment of FB and FH like a typical peptidomimetic compound, inhibiting the downstream AP complement processing, amplification, and negative-feedback regulation.

## Discussion

Viruses are known to adopt strategies at multiple levels of host cell immune regulation to thwart detection (8, 9, 10, 23, 24). The evasion mechanism of the innate immune surveillance system, consisting of a very robust complement network of the host often described as first line of defense against viruses, has not been demonstrated for Covid-19. Though multiple work described the activation mechanisms of the complement pathways leading to devastating consequences in covid-19–infected patients and hypothesized complement escape (25, 26), none seems to highlight the complement evasion strategies that the virus adopted to survive in the host during the initial phase of the infection cycle. We provide evidence that SARS-CoV-2 ORF8 protein plays a major role in chocking AP by interacting with C3/C3b and shielding major complement factors from binding. ORF8 interacts with C3b in such an optimized conformation that it not only prevents proteolysis by FI but also hinders activation of FB for functional C3-convertase formation. By cleaving C3b, FI hastens the decay of C3-convertase formation preventing hyperactivation and tissue damage. The binding of ORF8 to C3b appears to precede the binding of FH and recruitment of FB (26), resulting in blocking some of these essential functions in AP and allowing the virus to escape the detection and clearance.

ORF8 has been shown to exist both as homodimer and monomer (13, 27). Our in-silico results (Fig. 4) predict the c-terminal portion of the monomeric region, containing antiparallel β-strands linked by a hairpin-loop structural feature (Fig. S3) to fold into the C3b central core of the β-chain. These interactions, mostly with MG3 domain of the β-chain, orient the remaining part of the ORF8 molecule to occupy the surface near the CUB domain perhaps blocking the FH-FI binding. A C3b-interacting protein CRIg (complement receptor of the Ig superfamily; also called V-set and Ig domain–containing protein 4) has been known to interact primarily with the β-chain and reported to act as an inhibitor of convertase activity (28). Quite intriguingly, the ORF8 protein appears to have sequence resemblance not only with CRIg (about 39% similarity; Fig. S3) but also with the c-terminal region of FI protein sequence (Fig. S3). The combinations of limited sequence resemblance with specific domains of known C3-convertase down-regulators like FI and CRIg perhaps allows it to get access to the central β-core of various conformational states of C3b, rendering it inaccessible to the cofactors FH and FB, as demonstrated experimentally.

Opsonization by C3b is believed to play a central role in the protection of host against pathogens and its clearance by the complement system. Hence, viral and bacterial pathogens have evolved strategies to effectively target C3b specifically to gain evolutionary advantage (24). However, these strategies seem to be microbe specific. Herpes virus, being the first classical example, uses one of the several glycoproteins (gC1) on the viral surface to bind with C3b resulting in its inactivation and accelerating the decay of C3-convertase activity (29). On the other hand, viruses like Nipah uses protease activity reminiscent of FI protease to enhance degradation of C3b, thus blocking the complement activation *via* reduced C3-convertase formation (30). In contrast, the staphylococcal complement inhibitor act as a competitive inhibitor occluding FH binding (31). Similarly, ORF8 of SARS-CoV-2 appears to target critical steps of AP by interacting with C3b. These steps include occlusion of FH as evident from FI inhibition (Fig. 2) and prevention of FB activation (Fig. 3*A*). Thus, ORF8 appears to function more like staphylococcal complement inhibitor in inhibiting AP.

To conclude, this study provides direct evidence of binding of SARS-CoV-2 ORF8 protein to complement components C3/C3b, primarily making them resistant to binding to other major cofactors required for activation and regulation of the AP pathway. Though several questions remain unanswered at this stage in the context of ORF8-mediated physiological regulation of complement C3 activation, this work provides a unique framework to unravel further insights into the role of ORF8 in subverting complement surveillance.

## Experimental procedures

### Cells and transfection

5 × 10^6^ HepG2 Cells (NCCS Pune Cell Repository) were cultured in Dulbecco’s modified Eagle’s Medium with 10% (vol/vol) fetal bovine serum, 1% penicillin/streptomycin, and 2 mM L- Glutamine. HepG2 seeded in 9.6 mm^2^ 6-well culture plates 1 day before transfection. After 16 to 18 h, culture becomes 70 to 80% confluent. Fifteen micrograms SARS-CoV-2 ORF8 Gene-tagged ORF plasmid (Origene) were transfected using Lipofectamine 3000 (Invitrogen) according to the manufacturer instructions. HepG2 cells were harvested for further processing after 48 h of transfection.

### Coimmunoprecipitation

Flag-tagged SARS-CoV-2 ORF8–transfected HepG2 cells (10^6^) were harvested, lysed, and used as sample for IP with either one μg anti-FLAG (Sigma) or anti-Rabbit Human Complement C3 polyclonal antibody (Cloud-clone corp) or anti-rabbit IgG antibody (for Controls) mixed with Protein-A-Sepharose beads. The IP proteins were washed, extracted in SDS-sample buffer, and identified on immunoblots using appropriate primary antibodies. The chemiluminescent images were acquired in Chemi-Doc system (Bio-Rad) and intensity of each band is quantified by ImageJ Software (https://imagej.nih.gov/ij/) (NIH).

### Cofactor activity assay

Cofactor activity for FI-mediated cleavage of C3b (sigma cat. No. 20480) was measured in fluid-phase assay as described before (11). Briefly, 0.8 μM (2 μg) C3b and 50 nM (50 ng) FI (Sigma, cat. No. C5938) with 150 nM (312 ng) FH (Sigma, cat. No. C5813) with varying concentrations (2 μg-125 ng) of commercially obtained SARS-CoV-2 ORF8 (Thermo Fisher Scientific, Cat. No.RP- 87666) protein in 15 μl PBS 7.4. The mixture was incubated for 7 min at 37 °C and reaction stopped by mixing and boiling with 4X-SDS-PAGE sample-loading buffer. The cleavage products were resolved on 12% SDS-PAGE and visualized by Coomassie Brilliant Blue staining. Stained bands are quantified by densitometry using ImageJ software (NIH).

### AP C3-Convertase assay

The effect of ORF8 on fluid phase AP C3-convertase activity was determined following published assay (26). In brief, 0.4 μM–purified C3 (Quidel, Cat. No. A401) was incubated in the presence or absence of 5 and 10 μM ORF8 (Thermo Fisher Scientific) or 0.4 μM FH in GVB (20 μl volume) at 37 °C for 15 min. To start the assay, 0.4 μM FB (Quidel, Cat. No. A408) and 0.04 μM FD (Quidel, Cat. No. A409) were added in the presence of 33 mM Mg-EGTA in a total volume of 30 μl in each tube. The reaction was stopped after 30 min by boiling in reduced sample buffer and the products were resolved by 10% SDS-PAGE for visualization using Coomassie blue staining.

### Protein preparation for in-silico studies

The 3D structures of C3b complex (PDB ID: 5O32) and ORF8 (PDB ID: 7JTL) were retrieved. Both structures were processed and prepared in Schrodinger’s Protein preparation wizard module through Maestro GUI (32) by considering the bond order, H-bond assignment, and addition of missing side chains and loops. The missing side chains and loops in X-ray diffraction were modeled using Prime module of Schrodinger (33). The structures were then optimized at pH 7 and minimized using OPLS 2005 force-field as described before (34).

### Protein-protein docking

Using Schrodinger’s BioLuminate module, prepared protein structures were docked to identify the probable residues contributing to noncovalent interactions, following published protocol [(35)]. Briefly, clusters were generated in BioLuminate PIPER program applying a Fast-Fourier Transform approach, where higher cluster size signifies stable docked complex. For each complex, about 70,000 rotations were performed for ligand (ORF8) over the receptor protein (C3b) to obtain a docked complex. For this study, we have procured a total of five complexes which are then analyzed for their interactions.

## Data availability

All data are contained within the main manuscript and supporting information.

## Supporting information

This article contains supporting information.

## Conflict of interest

The authors declare that they have no conflicts of interest with the contents of this article.

## References

[1] Douglas S.R., Smith W. (1930). A study of vaccinal immunity in rabbits by means of *in vitro* methods. Br. J. Exp. Path.

[2] Java A., Apicelli A.J., Liszewski M.K., Coler-Reilly A., Atkinson J.P., Kim A.H. (2020). The complement system in COVID-19: friend and foe?. JCI Insight.

[3] Yu J., Gerber G.F., Chen H., Yuan X., Chaturvedi S., Braunstein E.M. (2022). Complement dysregulation is associated with severe COVID-19 illness. Haematologica.

[4] Holter J.C., Pischke S.E., de Boer E., Lind A., Jenum S., Holten A.R. (2020). Systemic complement activation is associated with respiratory failure in COVID-19 hospitalized patients. Proc. Natl. Acad. Sci. U. S. A..

[5] Afzali B., Noris M., Lambrecht B.N., Kemper C. (2022). The state of complement in COVID-19. Nat. Rev. Immunol..

[6] Lynch N.J., Chan A.C.Y., Ali Y.M., Khatri P., Bamigbola I.E., Demopulos G. (2022). Inhibition of the lectin pathway of complement ameliorates hypocomplementemia and restores serum bactericidal activity in patients with severe COVID-19. Clin. Transl. Med..

[7] Stoermer K.A., Morrison T.E. (2011). Complement and viral pathogenesis. Virology.

[8] Kotwal G.J., Isaacs S.N., McKenzie R., Frank M.M., Moss B. (1990). Inhibition of the complement cascade by the major secretory protein of vaccinia virus. Science.

[9] Kikkert M. (2020). Innate immune evasion by human respiratory RNA viruses. J. Innate Immun..

[10] Agrawal P., Nawadkar R., Ojha H., Kumar J., Sahu A. (2017). Complement evasion strategies of viruses: an overview. Front Microbiol..

[11] Kumar J., Yadav V.N., Phulera S., Kamble A., Gautam A.K., Panwar H.S. (2017). Species specificity of vaccinia virus complement control protein for the Bovine classical pathway is governed primarily by direct interaction of its acidic residues with factor I. J. Virol..

[12] Ricklin D., Reis E.S., Mastellos D.C., Gros P., Lambris J.D. (2016). Complement component C3 - the “Swiss Army Knife” of innate immunity and host defense. Immunol. Rev..

[13] Flower T.G., Buffalo C.Z., Hooy R.M., Allaire M., Ren X., Hurley J.H. (2021). Structure of SARS-CoV-2 ORF8, a rapidly evolving immune evasion protein. Proc. Natl. Acad. Sci. U. S. A..

[14] Xue X., Wu J., Ricklin D., Forneris F., Di Crescenzio P., Schmidt C.Q. (2017). Regulator-dependent mechanisms of C3b processing by factor I allow differentiation of immune responses. Nat. Struct. Mol. Biol..

[15] Roversi P., Johnson S., Caesar J.J., McLean F., Leath K.J., Tsiftsoglou S.A. (2011). Structural basis for complement factor I control and its disease-associated sequence polymorphisms. Proc. Natl. Acad. Sci. U. S. A..

[16] Wu J., Wu Y.Q., Ricklin D., Janssen B.J., Lambris J.D., Gros P. (2009). Structure of complement fragment C3b-factor H and implications for host protection by complement regulators. Nat. Immunol..

[17] Sahu A., Isaacs S.N., Soulika A.M., Lambris J.D. (1998). Interaction of vaccinia virus complement control protein with human complement proteins: factor I-mediated degradation of C3b to iC3b1 inactivates the alternative complement pathway. J. Immunol..

[18] Fromell K., Adler A., Aman A., Manivel V.A., Huang S., Duhrkop C. (2020). Assessment of the role of C3(H2O) in the alternative pathway. Front Immunol..

[19] Yu J., Yuan X., Chen H., Chaturvedi S., Braunstein E.M., Brodsky R.A. (2020). Direct activation of the alternative complement pathway by SARS-CoV-2 spike proteins is blocked by factor D inhibition. Blood.

[20] Milder F.J., Gomes L., Schouten A., Janssen B.J., Huizinga E.G., Romijn R.A. (2007). Factor B structure provides insights into activation of the central protease of the complement system. Nat. Struct. Mol. Biol..

[21] Fishelson Z., Pangburn M.K., Muller-Eberhard H.J. (1984). Characterization of the initial C3 convertase of the alternative pathway of human complement. J. Immunol..

[22] Geisbrecht B.V., Lambris J.D., Gros P. (2022). Complement component C3: a structural perspective and potential therapeutic implications. Semin. Immunol..

[23] Ploegh H.L. (1998). Viral strategies of immune evasion. Science.

[24] Sinha A., Singh A.K., Kadni T.S., Mullick J., Sahu A. (2021). Virus-encoded complement regulators: current status. Viruses.

[25] Charitos P., Heijnen I., Egli A., Bassetti S., Trendelenburg M., Osthoff M. (2021). Functional activity of the complement system in hospitalized COVID-19 patients: a prospective cohort study. Front Immunol..

[26] Forneris F., Ricklin D., Wu J., Tzekou A., Wallace R.S., Lambris J.D. (2010). Structures of C3b in complex with factors B and D give insight into complement convertase formation. Science.

[27] Matsuoka K., Imahashi N., Ohno M., Ode H., Nakata Y., Kubota M. (2022). SARS-CoV-2 accessory protein ORF8 is secreted extracellularly as a glycoprotein homodimer. J. Biol. Chem..

[28] Wiesmann C., Katschke K.J., Yin J., Helmy K.Y., Steffek M., Fairbrother W.J. (2006). Structure of C3b in complex with CRIg gives insights into regulation of complement activation. Nature.

[29] Fries L.F., Friedman H.M., Cohen G.H., Eisenberg R.J., Hammer C.H., Frank M.M. (1986). Glycoprotein C of herpes simplex virus 1 is an inhibitor of the complement cascade. J. Immunol..

[30] Johnson J.B., Borisevich V., Rockx B., Parks G.D. (2015). A novel factor I activity in Nipah virus inhibits human complement pathways through cleavage of C3b. J. Virol..

[31] Ricklin D., Tzekou A., Garcia B.L., Hammel M., McWhorter W.J., Sfyroera G. (2009). A molecular insight into complement evasion by the staphylococcal complement inhibitor protein family. J. Immunol..

[32] Sastry G.M., Adzhigirey M., Day T., Annabhimoju R., Sherman W. (2013). Protein and ligand preparation: parameters, protocols, and influence on virtual screening enrichments. J. Comput. Aided Mol. Des..

[33] Jacobson M.P., Pincus D.L., Rapp C.S., Day T.J., Honig B., Shaw D.E. (2004). A hierarchical approach to all-atom protein loop prediction. Proteins.

[34] Jorgensen W.L., Maxwell D.S., Tirado-Rives J. (1996). Development and testing of the OPLS all-atom force field on conformational energetics and properties of organic liquids. J. Am. Chem. Soc..

[35] Kumar P., Kumar A., Garg N., Giri R. (2021). An insight into SARS-CoV-2 membrane protein interaction with spike, envelope, and nucleocapsid proteins. J. Biomol. Struct. Dyn..

