# Supplementary material for: SARS-CoV-2–encoded ORF8 protein possesses complement inhibitory properties
Source: J Biol Chem. 2023 Jan 20;299(3):102930. doi: 10.1016/j.jbc.2023.102930 (PMC9851726; doi:10.1016/j.jbc.2023.102930)
Supplement: Supplemental Figures S1–S3 and Table S1 [file mmc1.pdf]

SARS-CoV-2-encoded ORF8 protein possesses complement inhibitory properties

Jitendra Kumar et al.

Supporting information on methods, figures and table

Experimental Methods

Preparation of gelatin veronal buffer (GVB)

Veronal buffer (5x concentration; Lonza, USA) was diluted with molecular biology grade water to prepare 1x concentration. GVB was prepared by dissolving gelatin (100 mM) in 1x veronal buffer by gentle warming.

Preparation of rabbit erythrocytes (rRBCs)

rRBCs were freshly collected from rabbits in 1:1 Alsever's solution (2.05% dextrose, 0.8% sodium citrate, 0.055% citric acid, and 0.42% sodium chloride) and kept on ice. 240 µl RBCs were spun down (~950g for 3 min at 4°C) and re-suspended in 1ml GVB. This process is repeated for multiple times till spontaneous haemolysis is stopped which is indicated by clear supernatant. To calculate the cell numbers, 10 µl RBCs were added to 240 µl of water and vortex to rupture RBCs. 100 µl volume from above suspension was transferred into microtiter plate (96 well, clear flat bottom) and absorbance was read at 405 nm in SpectroMax (Molecular Devices). Absorbance of 1.0 was considered to be equivalent to $1x10^9$ rRBC per millilitre

Normal human serum-induced hemolysis and hemolysis inhibition assay

For hemolysis assay, 10 µl of rRBC ($1x10^9$ cells) were added to 36 µl of GVB on ice. The hemolysis was initiated with the addition of 4 µl of normal human sera (NHS). The 50 µl of reaction mixture was then incubated for 20 min at 37°C. The lysis was stopped by addition

of 200 µl of 10 mM EDTA in GVB (GVBE solution) and centrifuged. 100 µl of the supernatant was collected for measuring the absorbance at 405 nm and the percentage of lysis was determined with reference to the absorbance of the reactant products in absence of NHS.

To determine SARS-CoV-2 encoded ORF8-mediated inhibition of the hemolysis, purified ORF8 protein was serially diluted from 5000 nM to 39 nM in a volume of 36 µl of GVB and pre-incubated for about 10 min with 10 µl of rRBC ($1x10^9$ cells). The percentage of inhibition of NHS-induced lysis at each concentration of ORF8 was calculated considering the lysis in absence of ORF8 as 100%.

Molecular Dynamics simulation analysis

The third ranked complex which is found to be in excellent correlation with the study, has been selected for the MD simulations studies. We have performed a 30 ns long MD simulation on this complex and along with the unbound (apo) proteins as well, in Gromacs simulation package. All solution systems with buffer condition were prepared on CHARMM-GUI webpage. Here, we have used TIP3P water model to solvate the system and Potassium (K^+) and Chloride (Cl^-) ions at a concentration of 0.15 M for charge neutralization. After preparing and obtaining the entire simulation setup, the setup was subjected to energy minimization of 5000 steps using steepest-descent method. Following energy minimization, the equilibration of 125000 steps at temperature 303.15 K and 1.0 bar pressure was carried out. Finally, a total of 30 ns long production run was executed using Nose-Hoover and Parrinello-Rahman algorithms for temperature and pressure coupling, respectively, on our in-house high performance cluster facility. The docked and simulated frames from trajectory was extracted and visualized for their interactions in Schrodinger’s Maestro GUI.

Figures and Legends

Figure S1: Characterization of 35 kDa C3d-like fragments (A) Image of the pre-stained protein ladder, Blueye from GeneDireX, USA (catalog number SP007-0500; Image from data sheet) with a molecular size range of 245 kDa -11 kDa as indicated. This marker was used for all figures except for Figure 2B in the main text. For Figure 2B, Rainbow-ladder, Amersham was used. (B) 1µg of C3 (Lane 1) was incubated with 330 ng of Factor H and 50 ng of factor I (Lane 2) along with 330 ng Trypsin in addition to C3, factor H, factor I (Lane 3) or Trypsin alone (Lane 4) in 15 µl of PBS 7.4 for 30 min at 37°C. Reaction mix were boiled with 5 µl of SDS-PAGE sample buffer for 5 min. 10% of the total reaction mix (2 µl) was analyzed for cleaved products by resolving on a 12% SDS-PAGE followed by immuno-detection by anti-C3 antibody (Cloud clone USA) on a western blot. A prominent 35 kDa band was detected in lane 3 and lane 4 (faint band in lane 2) indicating that the major 35 kDa C3d like fragment is generated from C3 via cleavage by Trypsin.

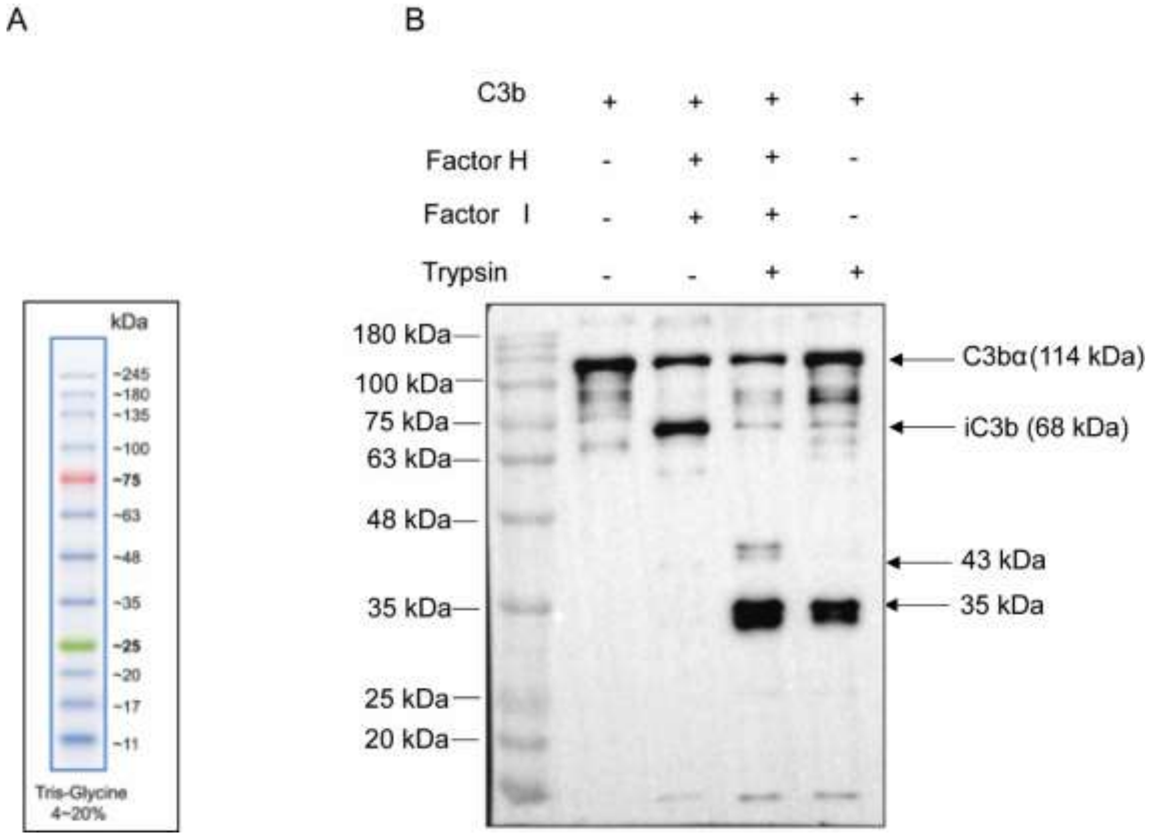

Figure S2: Docking of ORF8 on C3b-miniFH complex. Complexes formed using C3b-mini FH-FI structure (PDB entry 5O32) template after removal of FI and docking ORF8 (PDB:7JTL), did not accommodate ORF8 into the pocket near the CUB domain. Representative top 5 ranked structures have ORF8 (Salmon) associating with regions of C3b remote to the CUB domain in presence of mini FH (purple).

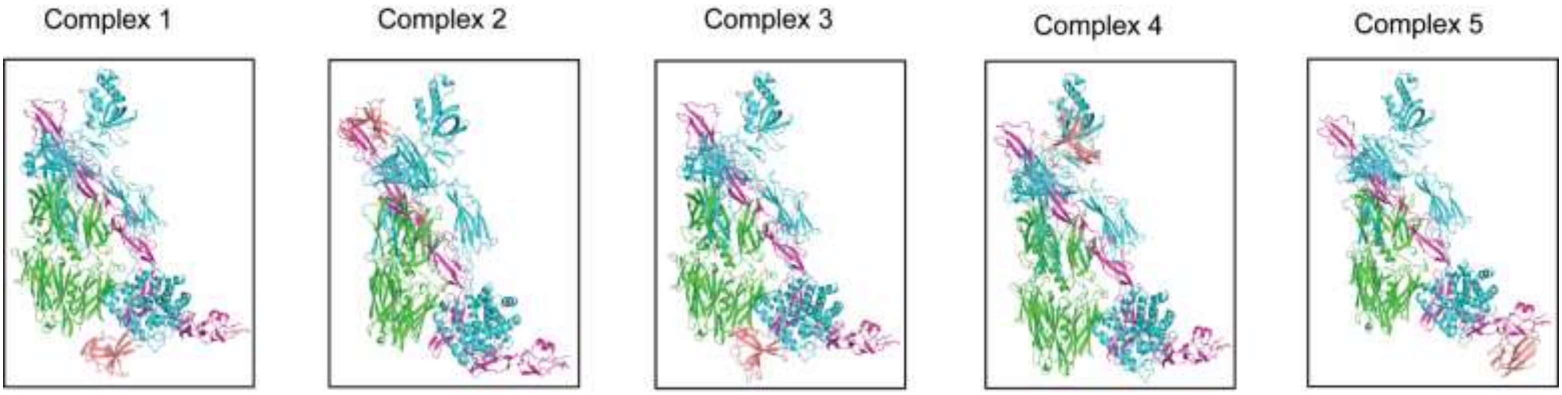

Figure S3: **A.** Protein Sequence alignment between human complement factor I (FI; GenBank: KAI 2535554.1) and SARS-CoV-2 ORF8 protein (Ref Sequence: YP_009724396.1) using Clustal Omega multiple sequence alignment program (EMBL-EBI tool service). Alignment numbering of FI according to GenBank: KAI2535554.1 (amino acid 506 – 591) and ORF8 following reference YP_009724396.1 (amino acid 40 – 121).

```
FI     GREKDNERVFSLQWGEVKLISNCSKFYGNRFYEKEMECAGTYDGSIDACKGDSG--GPLV
ORF8   -------------------------HFYSKWYIRVG--ARKSAPLIELCVDEAGSKSPIQ
                                .: .::* :    * .    *: * .::*  .*:

       CMDANNVTYVWGVVSWGENCGKPEFPGVYTKVANYFDWISYHVGRPFISQYNV       591
       YIDI--GNYTVSCLPFTINCQEPKLGSLVVRCSFYEDFLEYHDVRVVLDFI--       121
        :*    .*. . : :  ** :*:: .: .: : * *::.**  * .:.
```

The sequence resemblance region of ORF8 and Factor I serine protease domain is characterized by antiparallel β-strands linked by a loop, similar to a β-hairpin motif corresponding to 96 to 119 amino acid residues of ORF8 and 532-552 of FI.

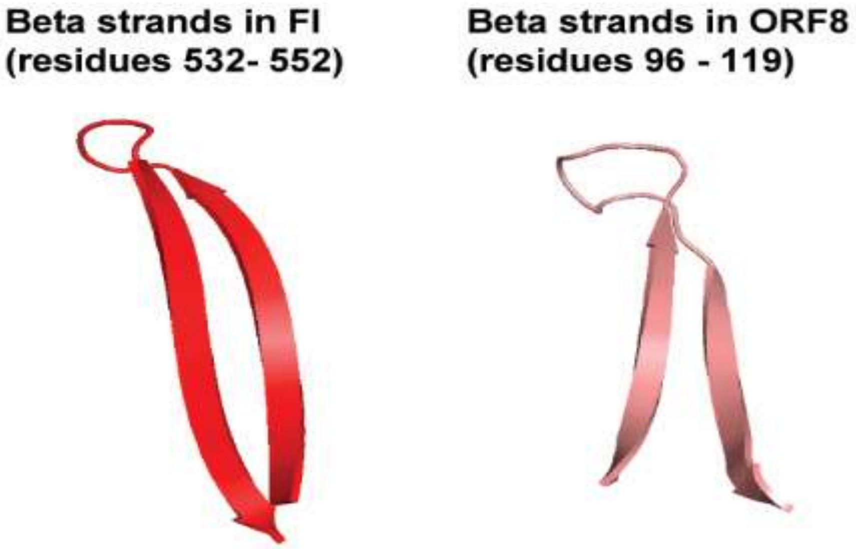

B. Protein Sequence alignment between human V-set and immunoglobulin domain-containing protein 4 (PDB entry 2ICC_1) and SARS-CoV-2 ORF8 protein (Ref Sequence: YP_009724396.1) using Clustal Omega multiple sequence alignment program (EMBL-EBI tool service).

```
2ICC_1|ChainA       -------GRP----ILE-----VPESVTGPWKGDVNLPCTYDPLQGYTQVLVKWLVQRGS      44
YP_009724396.1      MKFLVFLGIITTVAAFHQECSLQSCTQHQPYV--VDDPCPI-------HFYSKWYIRVGA      51
                           *       :.        :   *:   *: **         :.  ** :: *:

2ICC_1|ChainA       DPVT---IFLRDSSGDHIQQ-AKYQGRLHVSHKVPGDVSLQLSTLEMDDRSHYTCEVTWQ      100
YP_009724396.1      RKSAPLIELCVDEAGSKSPIQYIDIGNYTVS--------------------CLPFTINCQ      91
                       :    :  *.:*.:        *.  **                         :. *

2ICC_1|ChainA       TPD-GNQVVRDKITE-------LRVQK---      119
YP_009724396.1      EPKLGSLVVRCSFYEDFLEYHDVRVVLDFI      121
                     *. *. *** .: *       :**
```

*** (asterisk)** indicates positions which have a single, fully conserved residue.

: (colon) indicates conservation between groups of strongly similar properties as below - roughly equivalent to scoring > 0.5 in the Gonnet PAM 250 matrix

. (period) indicates conservation between groups of weakly similar properties as below - roughly equivalent to scoring =< 0.5 and > 0 in the Gonnet PAM 250 matrix.

Supporting Information Table S1

Interaction analysis of C3b-ORF8 docked complex. The interaction types shown in the last column are as follows: AHB: Aromatic Hydrogen Bond, HB: Hydrogen Bond.

**Docked complex**	**ORF8**	**C3B (chain ID)**	**Distance (Å)**	**Interaction type**
C3b-ORF8	HIS40	ASN939 (α)	1.72	HB
	HIS40	ASN939 (α)	2.57	AHB
	PHE104	GLU171 (β)	2.66	HB
	ASP119	TYR325 (β)	2.67	AHB
	ASP119	ARG281 (β)	2.56	HB
